# Biofilm Removal by Reversible Shape Recovery of the Substrate

**DOI:** 10.1021/acsami.0c20697

**Published:** 2021-04-06

**Authors:** Sang Won Lee, Joseph Carnicelli, Dariya Getya, Ivan Gitsov, K. Scott Phillips, Dacheng Ren

**Affiliations:** † Department of Biomedical and Chemical Engineering, Syracuse University, Syracuse, New York 13244, United States; ‡ Department of Chemistry, State University of New York - College of Environmental Science and Forestry, Syracuse, New York 13210, United States; § The Michael M. Szwarc Polymer Research Institute, Syracuse, New York 13210, United States; ∥ Center for Devices and Radiological Health, Office of Science and Engineering Laboratories, Division of Biology, Chemistry, and Materials Science, United States Food and Drug Administration, Silver Spring, Maryland 20993, United States; ⊥ Department of Civil and Environmental Engineering, Syracuse University, Syracuse, New York 13244, United States; # Department of Biology, Syracuse University, Syracuse, New York 13244, United States

**Keywords:** biofilm removal, shape memory polymer, dynamic surface topography, reversible, antibiotic susceptibility

## Abstract

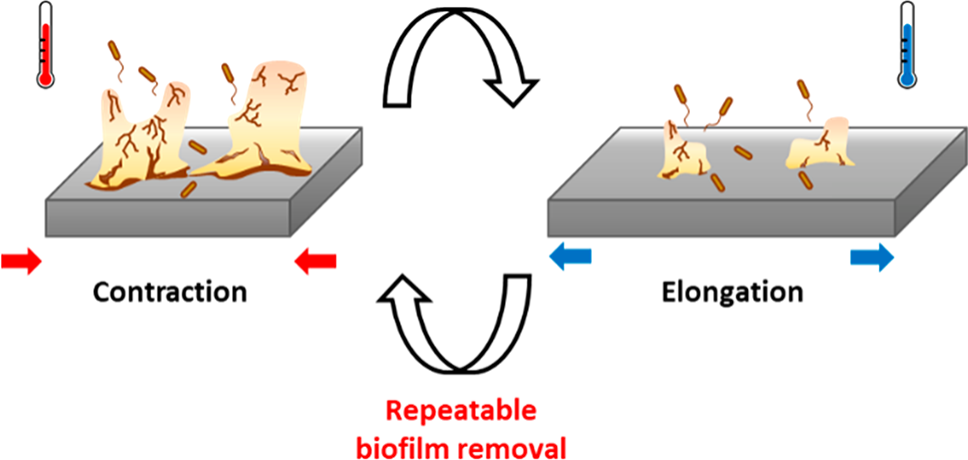

Bacteria can colonize essentially any surface and form antibiotic resistant biofilms, which are multicellular structures embedded in an extracellular matrix secreted by the attached cells. To develop better biofilm control technologies, we recently demonstrated that mature biofilms can be effectively removed through on-demand shape recovery of a shape memory polymer (SMP) composed of *tert-*butyl acrylate (tBA). It was further demonstrated that such a dynamic substratum can sensitize the detached biofilm cells to antibiotics. However, this SMP can undergo shape change only once, limiting its application in long-term biofilm control. This motivated the present study, which aimed to prove the concept that biofilm can be effectively removed by repeated on-demand shape recovery. Reversible shape memory polymers (rSMPs) containing poly(ε-caprolactone) diisocyanatoethyl dimethacrylate (PCLDIMA) of varying molecular masses and butyl acrylate (BA) as a linker were synthesized by using benzoyl peroxide (BPO) as a thermal initiator. By comparison of several combinations of PCLDIMA of different molecular masses, a 2:1 weight ratio mixture of 2000 and 15000 g/mol PCLDIMA was the most promising because it had a shape transition (at 36.7 °C) close to body temperature. The synthesized rSMP demonstrated good reversible shape recovery and up to 94.3 ± 1.0% removal of 48 h *Pseudomonas aeruginosa* PAO1 biofilm cells after three consecutive shape recovery cycles. Additionally, the detached biofilm cells were found to be 5.0 ± 1.2 times more susceptible to 50 μg/mL tobramycin than the static control.

## 1 Introduction

Microorganisms can attach to essentially any surface and develop multicellular structures known as biofilms. Because of the complex 3D structure and protective extracellular matrix, biofilms enable microbes to survive under challenging conditions including antimicrobial agents and host immune systems.^1^ The slow growth of bacterial cells in mature biofilms further contributes to the ineffectiveness of antibiotics, making mature biofilms extremely difficult to control.^2,3^ Although modern technologies have gradually reduced healthcare-associated infection (HAI) rates in the past decades,^4^ chronic infections associated with biofilms remain a major challenge in medicine.

This challenge has triggered intensive research on antifouling strategies. A common approach is coating the surface with antimicrobials or creating materials that release antimicrobials to kill bacterial cells directly.^5,6^ However, these methods are generally ineffective against mature biofilms due to their intrinsic antimicrobial tolerance. In addition, the broad application of antimicrobials may further promote the development of resistant strains. Alternatively, physical means have been explored to modify surface properties such as charge, hydrophobicity, stiffness, and topography.^7−13^ Unfortunately, most methods developed to date are limited to short-term *in vitro* conditions. Long-term infection control is hard to achieve because of the short duration of antimicrobial protection and the capability of biofilm bacteria to overcome unfavorable conditions.^14,15^

Dynamic surface topographies have been studied recently as a new approach to remove mature biofilms (see ref (16) for a recent review). Epstein et al.^17^ demonstrated up to 80% removal of 24 h *Pseudomonas aeruginosa* biofilm from PDMS surfaces by creating 2 μm dynamic wrinkles with uniaxial mechanical strain. Pneumatic actuation,^18^ electrical voltage,^19^ magnetic field,^20^ and air pressure^21^ were also used as a means to change a surface and remove established biofilms. In a previous study, we achieved on-demand biofilm control using *tert-*butyl acrylate (tBA)-based shape memory polymer (SMP) which demonstrated 99.9% removal of 48 h *Pseudomonas aeruginosa* biofilm compared to the static control.^22^ In addition, the cells detached by dynamic topography were sensitized to antibiotics.^23^ However, one of the major limitations of one-way SMP is that these materials can undergo shape change only once, which hampers the applications that require repeated actuation for long-term protection.

To prove the concept that it is possible to achieve fouling control with repeated actuation,^24^ an ε-caprolactone (ε-CL)-based copolymer cross-linked between two different molecular weights (low and high) was tested in this study. The melting temperature of copolymers was adjusted by changing the combination of ε-CL with different molecular masses. A reversible shape memory polymer (rSMP) with melting temperature around body temperature was chosen, and the shape recovery performance was investigated for its effects on biofilm removal and antibiotic susceptibility of the detached cells. The results demonstrated accumulative biofilm detachment with repeated shape recovery. Further research on this approach may provide better biomaterials for fouling control including those for safer medical devices.

## 2 Materials and Methods

### 2.1 Copolymer Synthesis

**Scheme 1:**
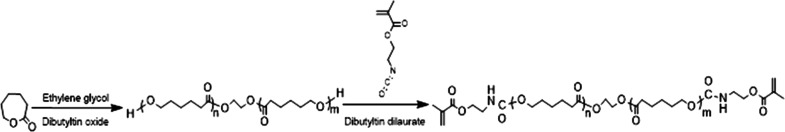
Synthesis of Poly(ε-caprolactone) Diisocyanatoethyl Dimethacrylate (PCLDIMA) by Ring-Opening Polymerization of ε-Caprolactone and Poly(ε-caprolactone) Diol, and Subsequent End-Group Modification with 2-Isocyanatoethyl Methacrylate

Poly(ε-caprolactone) diols (PCLs) were synthesized as described previously^24^ through a ring-opening polymerization (Scheme 1) using ε-CL (97%, Sigma-Aldrich, St. Louis, MO) and 1,2-dichloroethane (99.8%, Sigma-Aldrich), with ethylene glycol (99.8%, Sigma-Aldrich) as an initiator and dibutyltin oxide as a catalyst (98%, Sigma-Aldrich). PCLs with molecular masses of 8000 and 15000 g/mol were synthesized, and oligomers with molecular masses of 400, 600, 2000, and 4000 g/mol were purchased (99.9%, Perstorp, Malmo, Sweden). Ethylene glycol and ε-CL were mixed at a molar ratio of 1:100, supplemented with 1 wt % catalyst (dibutyltin oxide), and heated to 130 °C under N_2_. The progress of polymerization for 8000 g/mol (2 h) and 15000 g/mol (5 h) PCLs was monitored by using size exclusion chromatography (SEC). The crude products were purified by open column chromatography using silica gel and tetrahydrofuran (THF) as an eluent.

Nuclear magnetic resonance spectrometry (NMR; Bruker 600 MHz spectrometer, Billerica, MA) was conducted on the synthesized product. ^1^H NMR (600 MHz, CDCl_3_) δ: 4.35 (t, 4H, *J* = 6.45 Hz, −C*H*_2_OH), 4.05 [m, 4H, −C*H*_2_OC(O)−], 3.62 (m, 4H, −C*H*_2_OC*H*_2_−), 2.28 [m, 4H, −C*H*_2_C(O)−], 1.63 (m, 8H, −C*H*_2_CH_2_C*H*_2_−), 1.37 ppm (q, 4H, −CH_2_C*H*_2_CH_2_−)^25^ (Scheme 1 and Figure S1). ^13^C NMR (150 MHz, CDCl_3_) δ: 173.86, 173.72, 173.67, 173.32; 64.26, 64.23; 62.71, 62.19; 34.34., 34.29, 34.23, 34.00, 33.57; 32.43; 28.46; 25.67, 25.64, 25.61, 25.59, 25.41, 24.79, 24.69, 24.57, 24.48 ppm (Figure S2). Fourier transform infrared spectroscopy (FT-IR, ATR) spectra were obtained by a Bruker Tensor 27 spectrophotometer (Bruker, Billerica, MA): 3369 (−OH), 2944, 2865 (−CH_2_−), 1722 (−C=O), 1161 cm^–1^ (−O–C−) (Figure S3).

For end-group functionalization of PCLs, PCLs and 2-isocyanatoethyl methacrylate (98%, Sigma-Aldrich) were mixed at a 2:1 molar ratio with supplementation of 30 ppm dibutyltin dilaurate (95%, Sigma-Aldrich) as catalyst (in 50 mL of dichloromethane).^26^ The reaction was conducted for 5 days at room temperature under N_2_. After synthesis, a precipitation in hexane/methanol/diethyl ether (18:1:1) was used to purify PCL diisocyanatoethyl dimethacrylate (PCLDIMA)^26^ (yield 88%). ^1^H NMR (600 MHz, CDCl_3_) δ: 5.7 (dd, 4H, *J* = 316.85 Hz, =C*H*_2_), 4.2 [t, 4H, *J* = 5.0 Hz, −C(O)OC*H*_2_CH_2_NH−], 4.03 [m, 4H, −C*H*_2_OC(O)−], 3.62 (m, 4H, −C*H*_2_OC*H*_2_−), 3.49 [m, 4H, C(O)OCH_2_C*H*_2_NH−], 2.3 [m, 4H, −C*H*_2_C(O)−], 2.18 (s, 6H, =C–C*H*_3_), 1.62 (m, 8H, −C*H*_2_CH_2_C*H*_2_−), 1.38 ppm (q, 4H, −CH_2_C*H*_2_CH_2_−) (Figure S4). ^13^C NMR (150 MHz, CDCl_3_) δ: 173.65; 64.26, 64.18; 34.34, 34.23, 34.00; 32.44, 31.03; 28.47; 25.65, 25.42; 24.80, 24.69, 24.57 ppm. FT-IR (ATR): 2944, 2866 (−CH_2_−), 1722 (−C=O), 1161 cm^–1^ (−O–C−) (Figure S5).

To obtain the reversible shape memory polymers (rSMPs), PCLDIMAs with two different molecular masses were cross-linked at 90 °C with butyl acrylate (BA; 99%, Sigma-Aldrich) by using 1 wt % of the thermal initiator benzoyl peroxide (BPO; 98%, Sigma-Aldrich) (yield 89.2%). ^1^H NMR (600 MHz, CDCl_3_) δ: 5.6 (dd, 4H, *J* = 316.85 Hz, =C*H*_2_), 4.25 [t, 4H, *J* = 6.45 Hz, −C(O)OC*H*_2_CH_2_NH−], 4.03 [m, 4H, −C*H*_2_OC(O)−], 3.62 (m, 4H, −C*H*_2_OC*H*_2_−), 3.49 [m, 4H, C(O)OCH_2_C*H*_2_NH−], 2.3 [m, 4H, −C*H*_2_C(O)−], 1.95 [s, 9H, −C–(C*H*_3_)_3_], 1.62 (m, 8H, −C*H*_2_CH_2_C*H*_2_−), 1.38 ppm (q, 4H, −CH_2_C*H*_2_CH_2_−) (Figure S6). ^13^C NMR (150 MHz, CDCl_3_) δ: 173.69, 129.95, 129.01; 64.29, 63.94; 34.26; 28.50; 25.68, 25.46; 24.72; 18.44 ppm (Figure S7). FT-IR (ATR): 2944, 2866 (−CH_2_−), 1722 (−C=O), 1161 cm^–1^ (−O–C−) (Figure S8).

### 2.2 Programmable rSMP Substrate Preparation

To demonstrate reversible shape recovery, flat rSMPs were fixed into a curved shape. Briefly, a flat rSMP was incubated at 60 °C for 10 min, and the curved shape was fixed by using a glass cylinder and tape. The tape-fixed rSMP was cooled to room temperature for 10 min to maintain its curved shape, and then the tape was removed. The shape recovery performance was tested between 0 and 40 °C with 10 min incubation at each temperature. After each cycle, pictures of the sample were taken by a digital camera to measure a degree of curvature and shape change.

For a stretched rSMP, the flat surface was cut into a dog bone shape and stretched gently (in 10 min) with 18% elongation at 60 °C by using a manual stretcher. Under fixation, the stretched rSMP was then cooled to room temperature for 10 min. To recover the programmed rSMP, it was incubated in 0.85 wt % NaCl solution at a low temperature (0 °C or room temperature) and then at a higher temperature (40 °C) for 10 min at each of the temperatures. These two incubation steps comprise a cycle of shape recovery. Shape recovery alone (in the absence of biofilms) and with biofilm removal were tested for five and three cycles, respectively. The lengths of the programmed samples were measured after each cycle by a digital caliper to characterize the shape recovery ratio.

### 2.3 Bacterial Strain and Medium

*Pseudomonas aeruginosa* (*P. aeruginosa*) PAO1^27^ was grown at 37 °C in Lysogeny broth (henceforth LB medium)^28^ consisting of 10 g/L NaCl, 10 g/L tryptone, and 5 g/L yeast extract (Thermo Fisher Scientific, Waltham, MA).

### 2.4 Biofilm Formation

To grow biofilms, rSMPs were sterilized by UV light exposure for 1 h on each side, and *P. aeruginosa* PAO1 was used to inoculate each biofilm culture in a Petri dish containing sterilized rSMP samples (three in each) to an optical density of 0.05 at 600 nm (OD_600_). The biofilm samples were cultured at room temperature for 48 h before a triggered shape recovery.

### 2.5 Biomass

The effects of biofilm removal were evaluated by using imaging analysis. First, the 48 h *P. aeruginosa* PAO1 biofilms were washed with 0.85 wt % NaCl solution three times and stained with the Live/Dead Baclight bacterial viability kit (Life Technologies, Carlsbad, CA) for 15 min. The stained biofilm cells were then imaged by using an upright fluorescence microscope (Axio Imager M1, Carl Zeiss Inc., Berlin, Germany). We quantified the biomass of biofilms by analyzing 3D Z-stack images by using COMSTAT.^29^ Three biological replicates were analyzed for each condition, and five different positions were randomly selected from each sample.

### 2.6 Antibiotic Susceptibility

The antibiotic susceptibility of biofilm cells was determined by following the same procedure described in our previous studies.^23,30^ Briefly, rSMPs with attached biofilm cells were washed three times with 0.85 wt % NaCl solution and transferred to a 40 °C prewarmed test tube containing 2 mL of 0.85 wt % NaCl solution. After incubation for 10 min, the sample was transferred to a test tube at room temperature containing 0.85 wt % NaCl solution. Three cycles of temperature change were applied. For the programmed rSMPs, biofilm cells detached by shape recovery were harvested upon the completion of the third cycle. Biofilm cells on flat rSMPs were harvested by bead-beating for 30 s by using 0.1 g of 0.1 mm zirconia/silica bead (BioSpec Products, Inc., Bartlesville, OK). To avoid the confounding effect of bead-beating, the same bead-beating was also applied to the biofilm cells detached by shape recovery. The harvested biofilm cells from both the programmed rSMP and the static control were then treated with 50 μg/mL tobramycin (Tokyo Chemical Industry Co., Tokyo, Japan) for 1 h at 37 °C and washed three times with 0.85 wt % NaCl solution. The washed samples were plated on LB agar plates to count colony forming units (CFU)^31^ and antibiotic susceptibility was determined by comparing them to the controls.

### 2.7 Thermal and Mechanical Properties

Differential scanning calorimetry (DSC) was performed with 3–5 mg samples in crimped aluminum pans on a DSC Q200 instrument (TA Instruments, Waters Corporation, Milford, MA). Thermal transitions were monitored in a typical heating–cooling–heating sequence in the range −100 to 100 °C at 10 °C/min and were measured by using TA Instruments Explorer Q200-1801 software (TA Instruments, Waters Corporation, Milford, MA).

### 2.8 Statistics

Means and standard errors were calculated from three biological replicates. SAS 9.1.3, Windows version (SAS, Cary, NC), was used for all statistical analyses. Data with *p* < 0.05 were considered statistically significant.

## 3 Results

### 3.1 rSMP Synthesis

PCLs with two polymerizable end groups were successfully formed by modifying PCLs with 2-isocyanatoethyl methacrylate. The SEC traces of PCLDIMA 2K and 15K showed a monomodal molecular mass distribution without oligomer contamination (Figure S9). To synthesize a rSMP copolymer, poly(ε-caprolactone) diisocyanatoethyl dimethacrylate (PCLDIMAs) with two different molecular masses had to be cross-linked with 25 wt % butyl acrylate (BA) and 1 wt % thermal initiator, benzoyl peroxide (BPO). The thermal transitions were adjusted by altering the combination of PCLDIMAs with two different molecular masses. Both PCLDIMA 2K and PCLDIMA 15K showed a single cold crystallization event, but two melting transitions at the second heating sequence indicated the presence of two crystalline phases (Figures S10 and S11). Materials with a wide range of melting temperatures were obtained with reversible shape recovery effects. For possible use of rSMPs in biomedical applications, the melting temperature was adjusted around body temperature. Among all combinations of copolymers tested (Table 1), two PCLDIMA molecular masses (2000 and 15000 g/mol) with a weight ratio of 2:1 were chosen to form the backbone of the shape memory polymer with 25 wt % BA added as a cross-linker. Interestingly, the rSMP formed by the two PCLDIMAs does not have a melting transition but a broad shallow glass transition between 0 and 45 °C (Figure S12).

**Table 1:** Melting Temperatures of Cross-Linked Copolymers Formed by PCLDIMAs of Different Molecular Masses and 25 wt % BA with 1 wt % BPO

	melting temperature (°C)					
*M*_w_ (g/mol)	400	600	2000	4000	8000	15000
400				24.5	47.1	
600				32.9	47.6	
2000						45.4
4000	24.5	32.9			45.5	53.5
8000	47.1	47.6		45.5		
15000			43.2 (1:1)	53.3		
			36.7 (2:1)			

### 3.2 Reversible Shape Recovery

**Figure 1:**
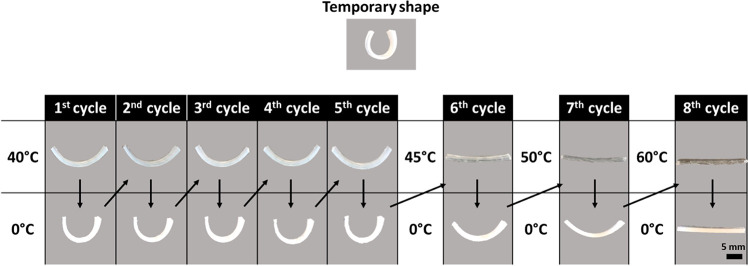
Reversible shape recovery of 2000 and 15000 g/mol (2:1 ratio) rSMPs (prepared from PCLDIMA with adding 25 wt % BA and 1 wt % BPO). Scale bar = 5 mm.

Based on the observed thermal transitions, 0 and 40 °C for repeated shape recovery were chosen first. The reversible shape recovery was conducted for five cycles first, and then the high temperature was gradually increased to 45, 50, and 60 °C in additional cycles to test the robustness of shape recovery. Figure 1 summarizes the shape recovery results. A temporary U shape of the rSMP was programmed and set as the initial state. At 40 °C, the rSMP deformed to a widely opened shape, as programmed, and it was deformed back into a slightly opened shape at 0 °C. After the first cycle of shape recovery, the rSMP was at a more open state than the initially programmed U shape presumably due to the specific balance between two polymer segments.^26,32^ However, both U shapes at 40 and 0 °C were well maintained after the fifth cycle. As the set high temperature increased by 5 °C after the fifth cycle, the rSMP gradually lost its shape. At 60 °C the surface became flat. This result is expected because the applied high temperature of 60 °C exceeded the range of melting transitions for programmed deformation.

### 3.3 Biofilm Removal by Reversible Shape Recovery

**Figure 2:**
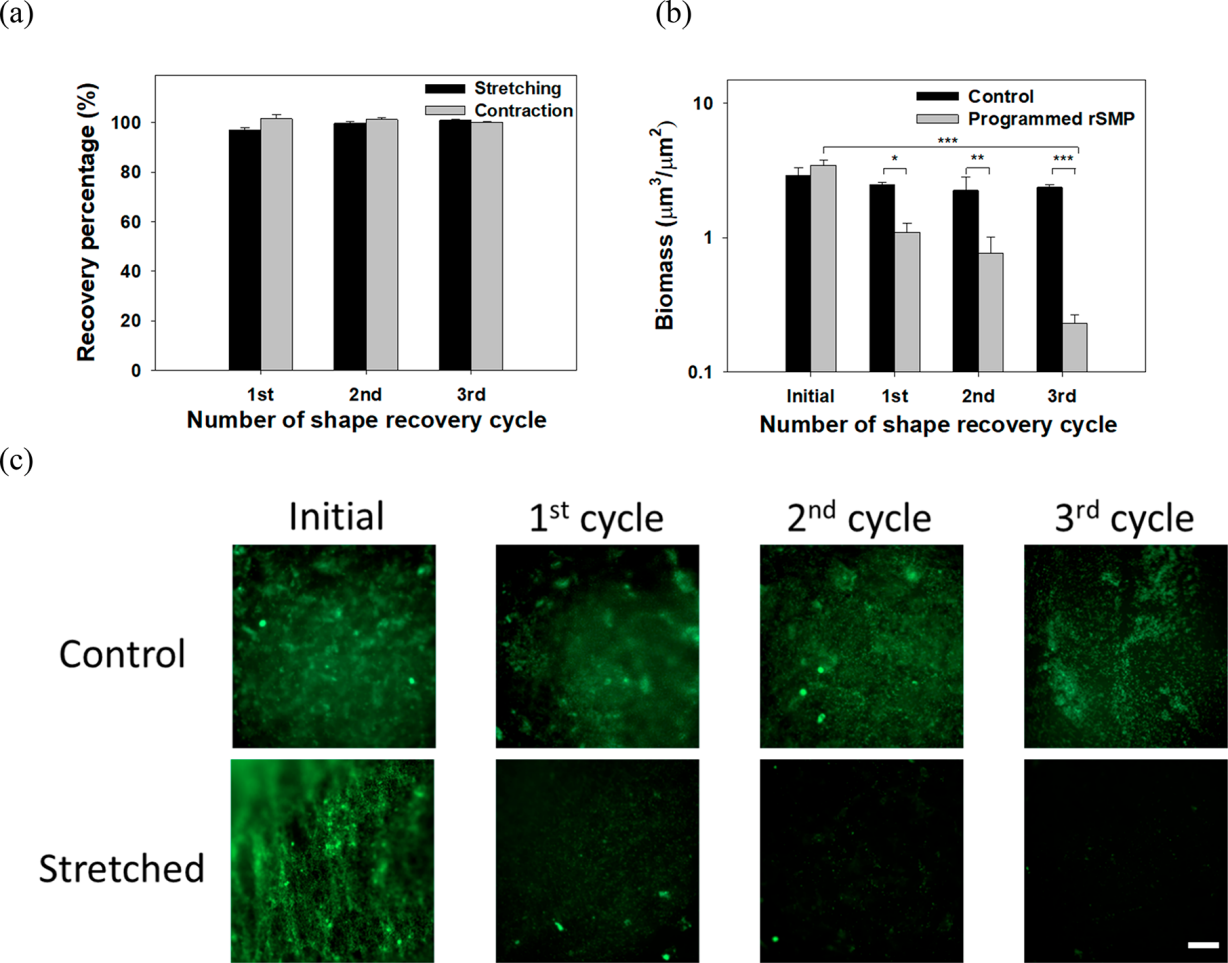
Shape recovery and biofilm removal between 0 and 40 °C. (a) Percentage of shape recovery of the synthesized rSMP after each cycle compared to the previous cycle. The first cycle was compared with the original dimension after programming. (b) Biomass after each cycle. (c) Representative biofilm images after Live/Dead staining (*p < 0.05, **p < 0.01, ***p < 0.001. *n* = 3). Scale bar = 100 μm.

After confirming repeated shape change, the biofilm removal was tested by stretching rSMPs bidirectionally with 18% elongation. *P. aeruginosa* PAO1 was cultured to form biofilms on UV-sterilized rSMP samples at room temperature for 48 h. Each cycle of shape recovery was conducted between 0 and 40 °C, and the biomass on the substratum was quantified by analyzing 3D Z-stack Live/Dead images using COMSTAT.^29^Figure 2a shows a good shape recovery behavior, e.g., 96.9 ± 1.0% at the end of three cycles (based on the length of rSMP samples). Consistently, the biomass of *P. aeruginosa* PAO1 was significantly reduced by shape recovery (Figure 2b). There was no significant change in biomass on static control (same material but without programming) after three cycles of shape recovery. In comparison, the biomass on the programmed rSMPs was 55.0 ± 6.1%, 77.6 ± 6.5%, and 93.6 ± 0.8% lower than the static control after the first, second, and third cycle of shape recovery (*p* = 0.004, 0.036, and 0.00004, *t* test), respectively. This corresponds to a total of 94.3 ± 1.0% biomass reduction after three cycles compared to the initial biomass (*p* < 0.001, one-way ANOVA followed by the Tukey test). The CFU results were corroborated by fluorescence microscopy, which showed a substantial reduction of surface coverage with no cell death observed based on Live/Dead staining (Figure 2c).

**Figure 3:**
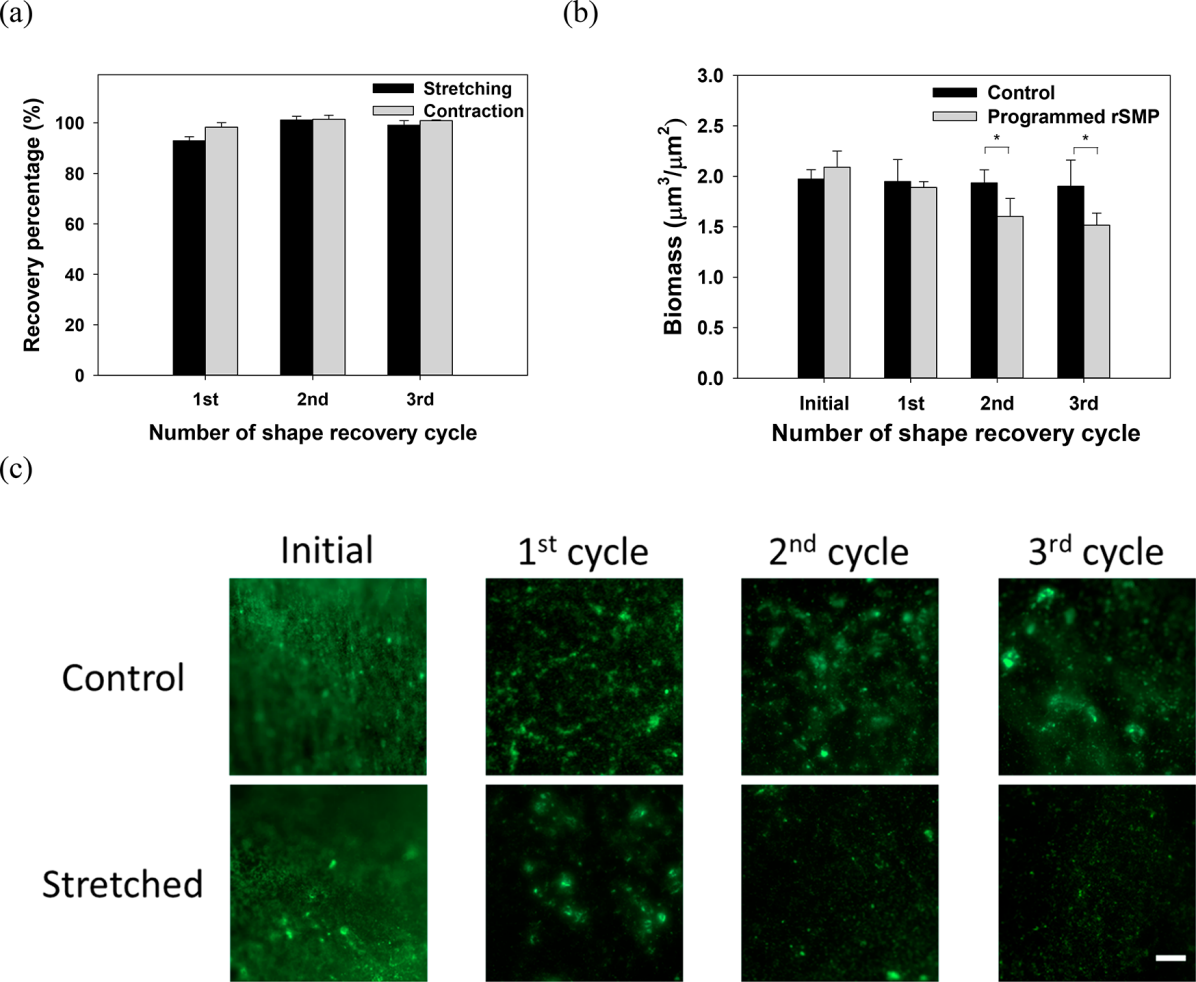
Shape recovery behavior and biofilm removal between RT and 40 °C. (a) Percentage of shape recovery of the synthesized rSMP after each cycle compared to the previous cycle. The first cycle was compared with the original dimension after programming. (b) Biomass after each cycle. (c) Representative biofilm images after Live/Dead staining. (*p < 0.05, *n* = 3). Scale bar = 100 μm.

The experiments above demonstrated the feasibility of additional biofilm removal using repeated shape recovery. However, 0 °C is rather harsh for many applications. Thus, biofilm removal was further tested between room temperature and 40 °C, and good shape recovery behavior was observed (98.9 ± 1.2% on average in three cycles, Figure 3a). The effects on biofilms were less pronounced than those between 0 and 40 °C, but significant biofilm removal was still achieved; e.g., 21.6 ± 1.7% (*p* = 0.014, *t* test) after three cycles of shape recovery (Figure 3b). Consistently, fluorescence microscopy of Live/Dead staining revealed biofilm removal with no cell death (Figure 3c). Because shape recovery occurred faster between 0 and 40 °C than between RT and 40 °C, a higher recovery rate may be favorable for biofilm removal.

### 3.4 Reproducibility of Biofilm Removal through Shape Recovery

**Figure 4:**
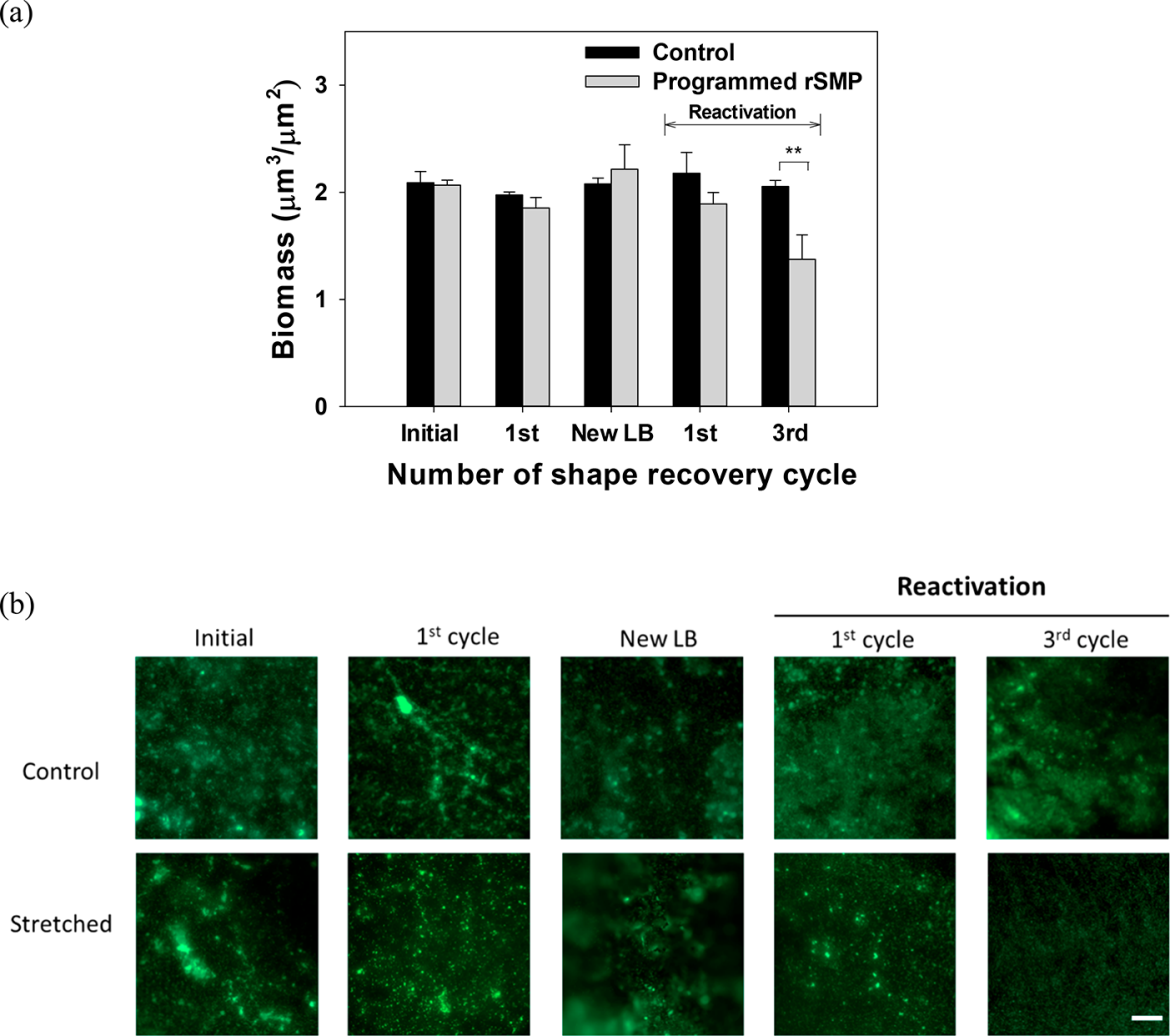
Shape recovery after biofilm regrowth (between RT and 40 °C). (a) Biomass after each cycle. After the first shape recovery, the sample was transferred into new LB medium and the biofilm was regrown for 2 days. Then, reactivation of shape recovery was triggered for three more cycles, and biomass was measured after the first and third cycles. (b) Representative images of biofilms. **p < 0.01 (*n* = 3). Scale bar = 100 μm.

Reversible shape recovery allows repeated on-demand actuation when biofilm builds up, bringing a possibility for long-term biofilm control. To test whether the strategy is still effective after biofilm regrowth, the rSMP was transferred into fresh LB medium, after the first shape recovery, to grow the remaining biofilm for 48 h at room temperature. As shown in Figure 4a, the biomass of remaining biofilm cells increased after further incubation in LB medium for 48 h. When shape recovery (between RT and 40 °C) was triggered after biofilm regrowth, significant biofilm removal was achieved after three consecutive cycles of shape recovery with a total of 32.8 ± 7.2% biomass reduction (*p* = 0.007, *t* test) compared to the static control. The CFU results were corroborated by fluorescence microscopy results with no cell death observed based on Live/Dead staining (Figure 4b). Thus, biofilm removal was achieved with reactivation of shape recovery after biofilm regrowth.

### 3.5 Biofilm Removal Sensitized Detached Cells to Tobramycin

**Figure 5:**
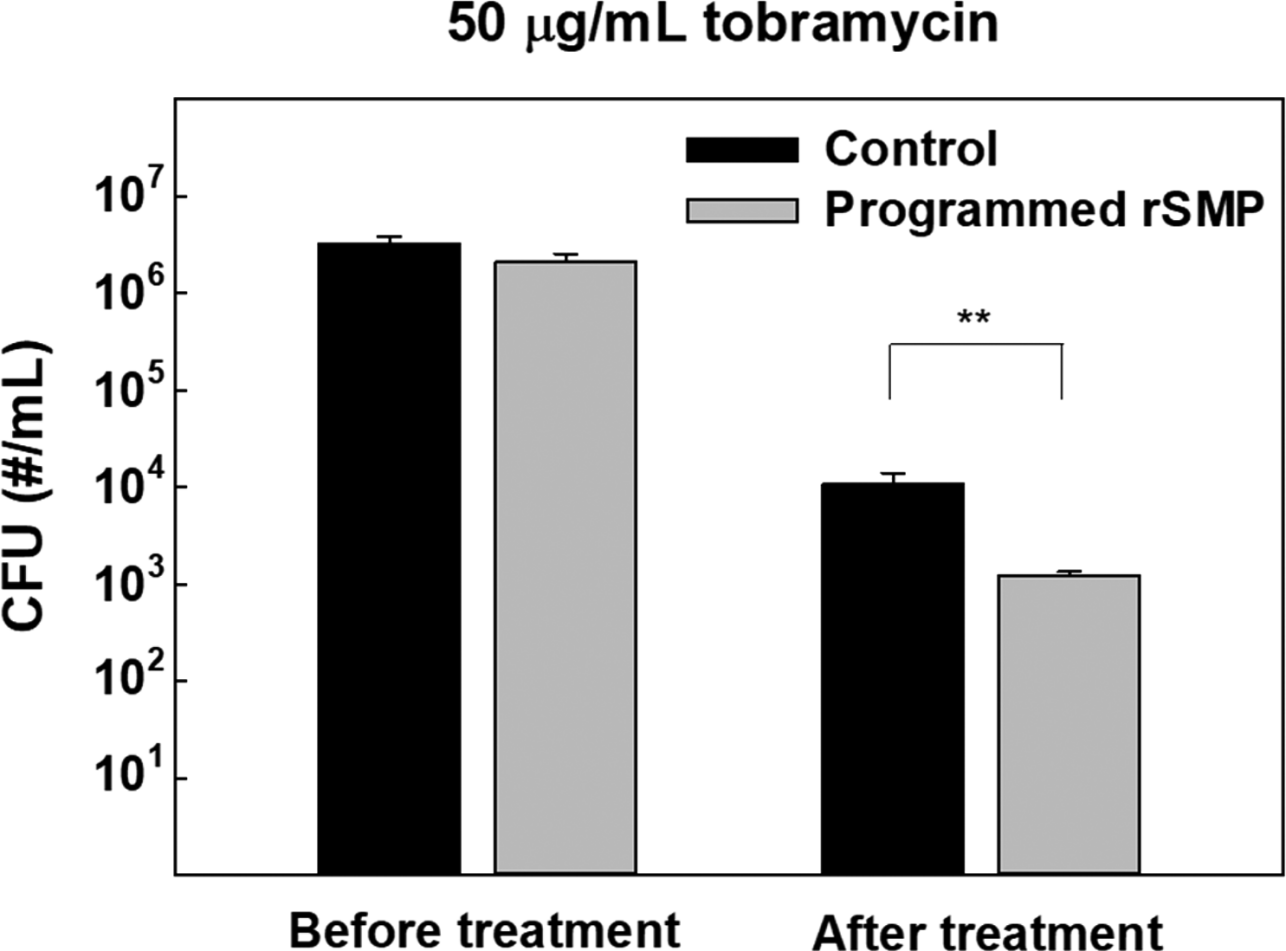
Detached *P. aeruginosa* PAO1 biofilm cells were more susceptible to tobramycin. Tobramycin at 50 μg/mL was tested by adding to the biofilm cells dispersed after the third shape recovery cycle. The biofilm cells of static flat control were detached by bead beating. The biofilm cells released by shape recovery were also processed with bead beating to avoid confounding effects (**p < 0.01, *n* = 3).

In a previous study, we demonstrated that the shape recovery of tBA based one-way SMP can sensitize the detached biofilm cells to antibiotics likely due to the increase in the intracellular level of ATP and metabolic activity of detached cells.^23^ To understand whether rSMP has similar effects, the tobramycin susceptibility of *P. aeruginosa* cells detached by shape recovery was evaluated and compared with control cells (detached by bead beating from static control surfaces). The cells detached by shape recovery were also processed with bead-beating to avoid confounding effects. As shown in Figure 5, the biofilm cells detached by shape recovery were 0.7 ± 0.1 log (5.0 ± 1.2 times) more susceptible to the 50 μg/mL tobramycin than the control (*p* = 0.004, *t* test). Thus, reversible shape recovery of the rSMPs can also sensitize detached biofilm cells to tobramycin. This is encouraging because biofilm cells are highly tolerant to antibiotics.

## 4 Discussion

Biofilms are blamed for numerous problems in both medical and industrial settings. With the efficacy of conventional antibiotics being limited, it is important to develop new methods to effectively remove mature biofilms and/or sensitize biofilm cells to antibiotics.

In a previous study, we demonstrated biofilm control using on-demand shape recovery of SMP, and 99.9% removal of mature (48 h) *P. aeruginosa* PAO1 biofilms was obtained.^22^ In addition, it was demonstrated that dynamic topography can sensitize the detached biofilm cells to antibiotics possibly due to the elevated intracellular ATP level and other related changes in cell physiology. Shape recovery induced biofilm detachment resulted in significant changes in gene expression, especially genes related to metabolic activities, which could render the cells to enter a more active stage and be more prone to antibiotics especially bactericidal antibiotics.^23^ However, one-way SMP cannot be activated repeatedly over time and thus may not be effective for long-term applications. To address this limitation, a ε-caprolactone-based SMP capable of reversible shape recovery was tested in this study. It showed a good shape recovery performance (98.9 ± 1.2% on average in three cycles) between room temperature and 40 °C. Under the same condition, 48 h *P. aeruginosa* PAO1 biofilm was removed by 21.6 ± 1.7% after three consecutive cycles. In a complementary experiment, the PAO1 biofilm after one shape recovery cycle was further incubated for 48 h in LB medium to regrow before another three cycles of shape recovery, which had 32.8 ± 7.2% biofilm removal compared to the static control. Moreover, a synergic effect between antibiotic treatment and biofilm removal was observed for the detached cells, showing 5.0 ± 1.2 times higher susceptibility to 50 μg/mL tobramycin compared to the control cells (detached from static control surfaces by bead beating). On the basis of the previous study of one-way shape recovery,^23^ we speculated that the intracellular ATP level and metabolic activity may be higher in the biofilm cells detached by reversible shape recovery of rSMP, leading to the increase in antibiotic susceptibility. This will be tested in our future work.

Several stimuli have been shown to trigger shape change of SMPs such as heat,^22^ solvent,^33^ electricity,^34^ and ultrasound.^35^ Heat has been the most commonly used trigger for biomedical applications. In this study, the synthesized rSMP is a chemically cross-linked semicrystalline polymer with heat-induced shape recovery.^26^ PCLDIMAs with two different molecular masses were cross-linked at 90 °C. The reversible shape memory effect requires a wide range of melting transitions.^32^ The two segments of the rSMP had two different melting temperatures. Thus, by varying the ratios of the two blocks, a wide range of melting transitions can be achieved. Within the wide melting temperature range, two elements coexisted as a “shifting-geometry determining segment” (an element with a higher melting temperature) and an “actuator segment” (an element with a lower melting temperature). After programming the rSMP, the stretched sample shrunk at high temperature when the crystalline phase of the “actuator segment” is partially melted, leading to an increase in contraction force. The sample was contracted to the intermediate deformation until the contraction force and an internal tensile force were balanced. At a low temperature, on the other hand, the internal tensile force becomes dominant, and this results in a further elongation of the rSMP. The specific system tested in this study allows 18% stretching under our experimental conditions. It is expected that the effects of biofilm removal can be stronger if the system is improved to allow more stretching, e.g., 99.9% removal in one step as demonstrated in our earlier study of a one-way SMP with 50% stretching.^22^ By use of the same principle of reversible shape recovery, other materials of copolymers with different melting temperature ranges have been described,^36^ which can be tested as future antifouling materials. Future research can also develop other rSMPs for better shape recovery and antifouling activities. This is part of our ongoing work.

**Figure 6:**
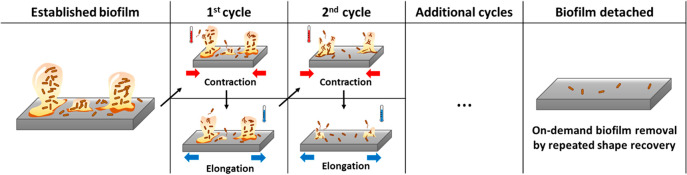
Schematic illustration of biofilm removal by reversible shape recovery. Biofilms can be removed by repeated recovery effects with contraction and elongation of the substrate rSMP material.

SMPs have been applied in the biomedical field such as self-tightening sutures,^37^ self-expansion stents,^38^ drug delivery carriers,^39^ and artificial bandages^40^ but mostly focused on one-way SMPs. Further development of reversible SMPs will help engineer novel materials/devices that are more programmable and responsive to disease factors and other stimuli. Although the triggering temperatures need to be further optimized, the results from this study proved the feasibility to obtain repeated actuation and biofilm removal. Figure 6 provides a summary of this approach. By coating the internal surface of medical devices such as catheters with rSMP material, it may be possible to engineering self-cleaning devices to prevent/treat device-associated infections.

The mechanism of biofilm removal also deserves further study. It is speculated that shape recovery can break the connection between a biofilm and the substrate surface, which triggers physiological changes in attached cells, leading to biofilm detachment and enhanced antibiotic susceptibility of detached cells. Further mechanistic study will provide important information for a better understanding of the potential of this technology for long-term biofilm control.

## 5 Conclusions

In summary, this study proved the concept of biofilm removal by repeated shape recovery of rSMPs. The rSMP in this study was synthesized with 2000 and 15000 g/mol PCLDIMA at a ratio of 2:1 with BA as a cross-linker and 1 wt % BPO as a thermal initiator. The shape memory effect of the rSMP can be preserved with good reproducibility over at least five cycles. Consistently, mature (48 h) *P. aeruginosa* PAO1 biofilms were efficiently removed (up to 94.3 ± 1.0%) after three cycles of consecutive shape recovery. Dynamic changes of the substrate material also sensitized the detached biofilm cells to tobramycin compared to the static control. With the capability of repeated shape recovery, rSMPs have potential applications for long-term biofilm control.

## Supporting Information Available

^1^H NMR and ^13^C NMR spectra of PCL (Figures S1 and S2), FT-IR analysis of PCL (Figure S3), ^1^H NMR spectra of PCLDIMA (Figure S4), FT-IR analysis of PCLDIMA (Figure S5), ^1^H NMR and ^13^C NMR spectra of rSMP (Figures S6 and S7), FT-IR analysis of rSMP (Figure S8), size-exclusion chromatography (SEC) of PCLDIMA precursors (Figure S9), differential scanning calorimetry (DSC) of 2K and 15K PCLDIMA and rSMP (Figures S10–S12) (PDF)

## Notes

**Disclaimer**: The mention of commercial products, their sources, or their use in connection with material reported herein is not to be construed as either an actual or implied endorsement of such products by the Department of Health and Human Services. The findings and conclusions in this paper have not been formally disseminated by the US Food and Drug Administration and should not be construed to represent any agency determination or policy.
