# Supplementary material for: Biofilm Removal by Reversible Shape Recovery of the Substrate
Source: ACS Appl Mater Interfaces. 2021 Apr 6;13(15):17174–82. doi: 10.1021/acsami.0c20697 (PMC8153534; doi:10.1021/acsami.0c20697)
Supplement: Supplementary file 1 — am0c20697_si_001.pdf [file am0c20697_si_001.pdf]

Supporting Information

Biofilm Removal by Reversible Shape Recovery of the Substrate

Sang Won Lee[1⊥], Joseph Carnicelli[1], Dariya Getya[2,3], Ivan Gitsov[2,3], K. Scott Phillips[4], and Dacheng Ren[1,5,6]*

[1]Department of Biomedical and Chemical Engineering, Syracuse University, Syracuse, NY 13244, United States

[2]Department of Chemistry, State University of New York - College of Environmental Science and Forestry, Syracuse, NY 13210, United States

[3]The Michael M. Szwarc Polymer Research Institute, Syracuse, NY 13210, United States

[4]United States Food and Drug Administration, Office of Medical Products and Tobacco, Center for Devices and Radiological Health, Office of Science and Engineering Laboratories, Division of Biology, Chemistry, and Materials Science, Silver Spring, MD 20993, United States

[5]Department of Civil and Environmental Engineering, Syracuse University, Syracuse, NY 13244, United States

[6]Department of Biology, Syracuse University, Syracuse, NY 13244, United States

*Corresponding author:

Dacheng Ren: Phone +1-315-443-4409. Fax +1-315-443-9175. Email: dren@syr.edu

[⊥]Present address:

Sang Won Lee

United States Food and Drug Administration, Office of Medical Products and Tobacco, Center for Devices and Radiological Health, Office of Science and Engineering Laboratories, Division of Biology, Chemistry, and Materials Science, Silver Spring, MD 20993

Contents

^{1}H NMR analysis of starting material (PCL) ………………………………………...……… S1

^{13}C NMR analysis of starting material (PCL) ………………………………………......... S2

FT-IR analysis of starting material (PCL) …………………………………………........... S3

^{1}H NMR analysis of starting material (PCLDIMA) ……………………………………..…S4

FT-IR analysis of starting material (PCLDIMA) ……………………………………………… S5

^{1}H NMR analysis of starting material (rSMP) ……………………………………...……...S6

^{13}C NMR analysis of starting material (rSMP) ……………………………………….........S7

FT-IR analysis of starting material (rSMP) ……………………………………………..........S8

Size-exclusion chromatography (SEC) of PCLDIMA precursors……………………………….S9

Differential scanning calorimetry (DSC) of PCLDIMA 2k……………………………………S10

Differential scanning calorimetry (DSC) of PCLDIMA 15k…………………………………..S11

Differential scanning calorimetry (DSC) of rSMP……………………………………………..S12

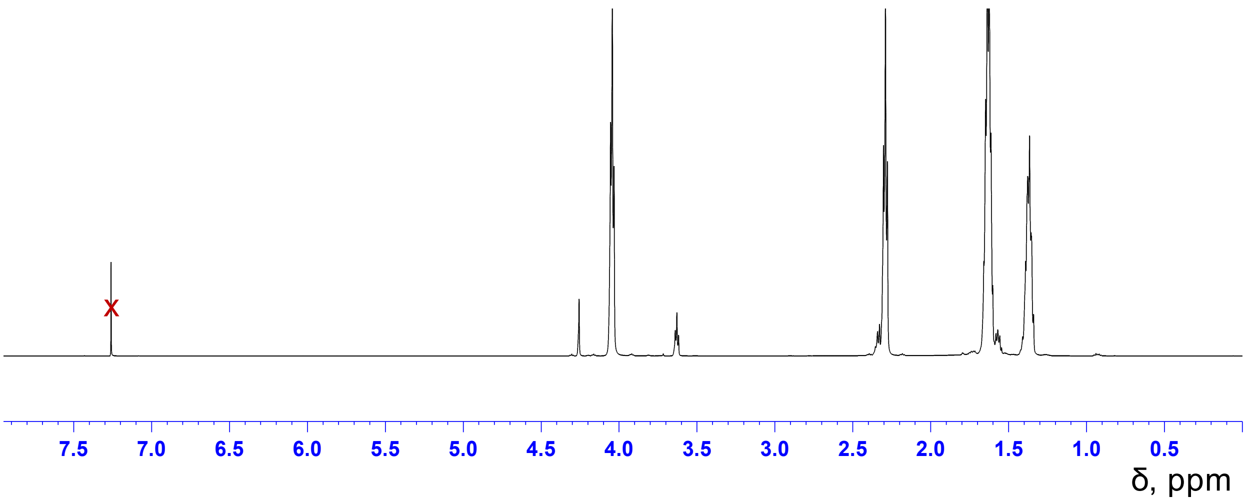

Figure S1. ^{1}H NMR spectrum of PCL 15k recorded at 600 MHz in $CDCl_3$ at room temperature. X – solvent signal.

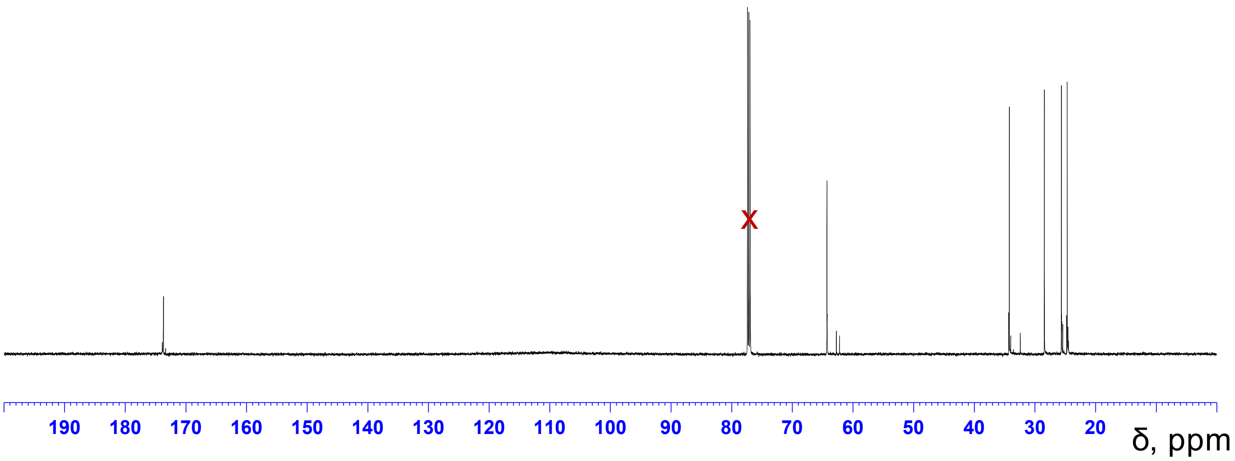

Figure S2. ^{13}C NMR spectrum of PCL 15k recorded at 152 MHz in $CDCl_3$ at room temperature. X – solvent signal.

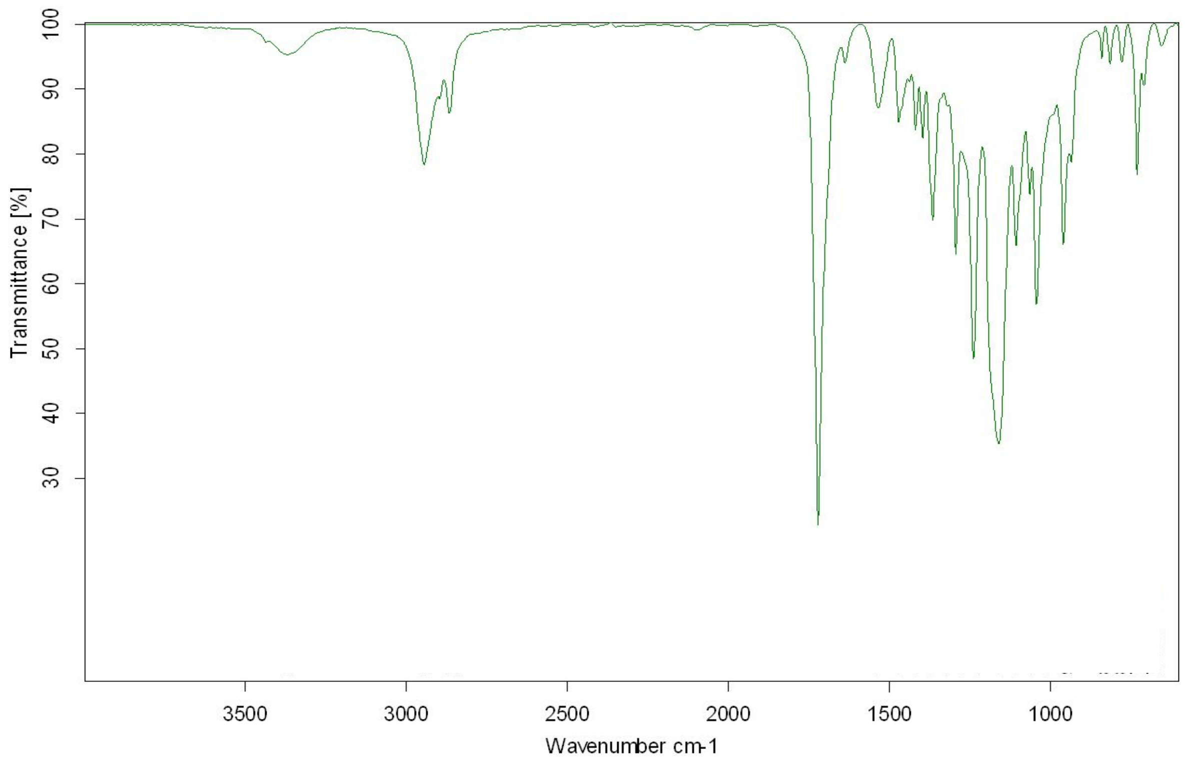

Figure S3. ATR FT-IR spectrum of PCL 15k.

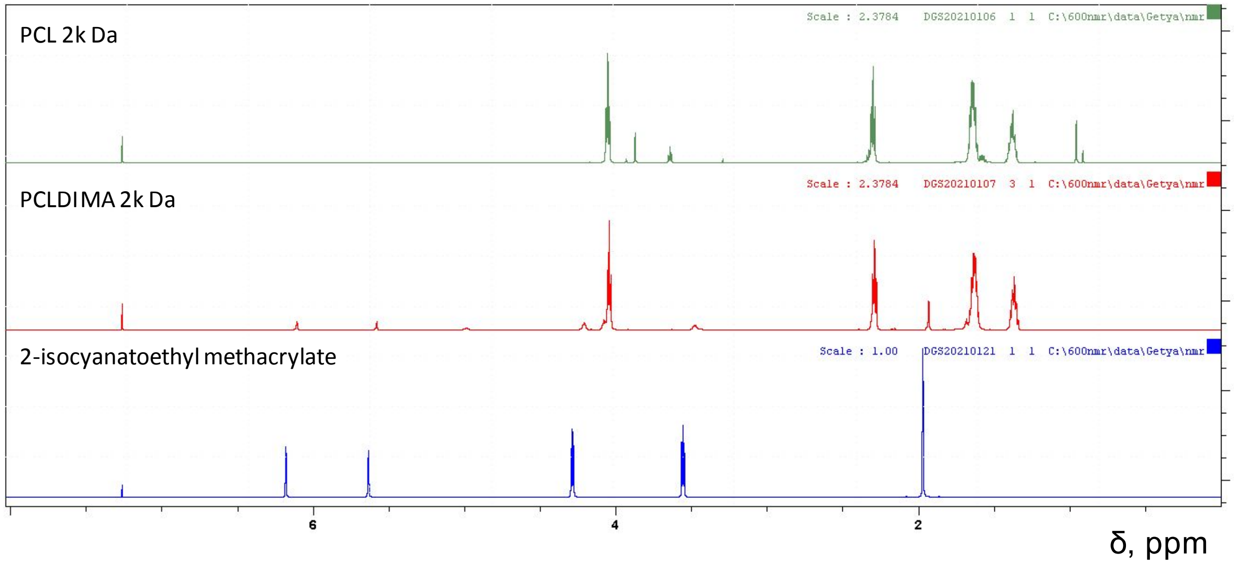

Figure S4. Overlaid 600 MHz ^{1}H NMR spectra of 2-isocyanatoethyl methacrylate (bottom, blue trace), PCLDIMA 2k (middle, red trace), and PCL2k (top, green trace).

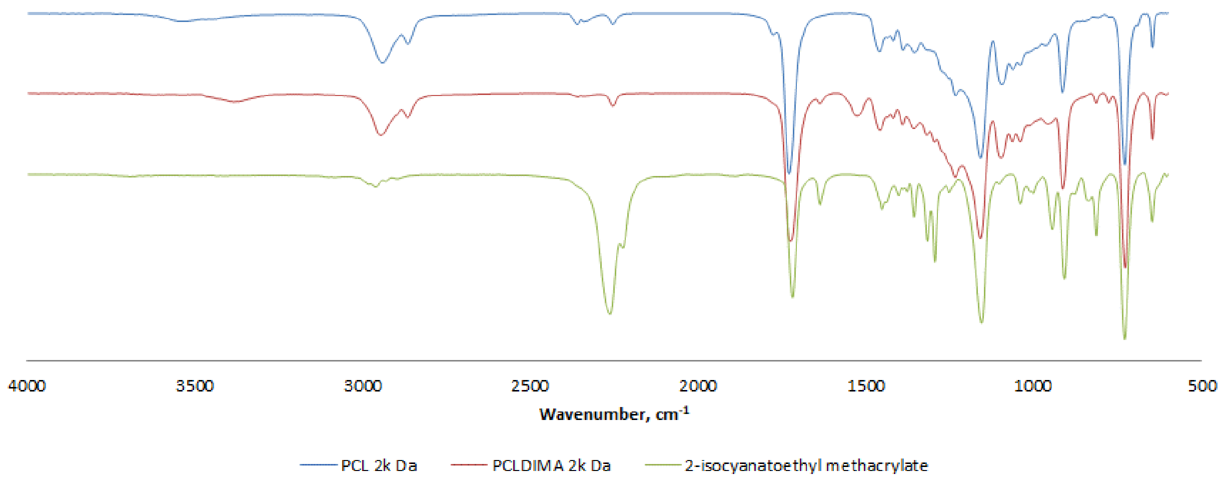

Figure S5. Overlaid ATR FT-IR spectra of 2-isocyanatoethyl methacrylate (bottom, green trace), PCLDIMA 2k (middle, red trace), and PCL2k (top, blue trace).

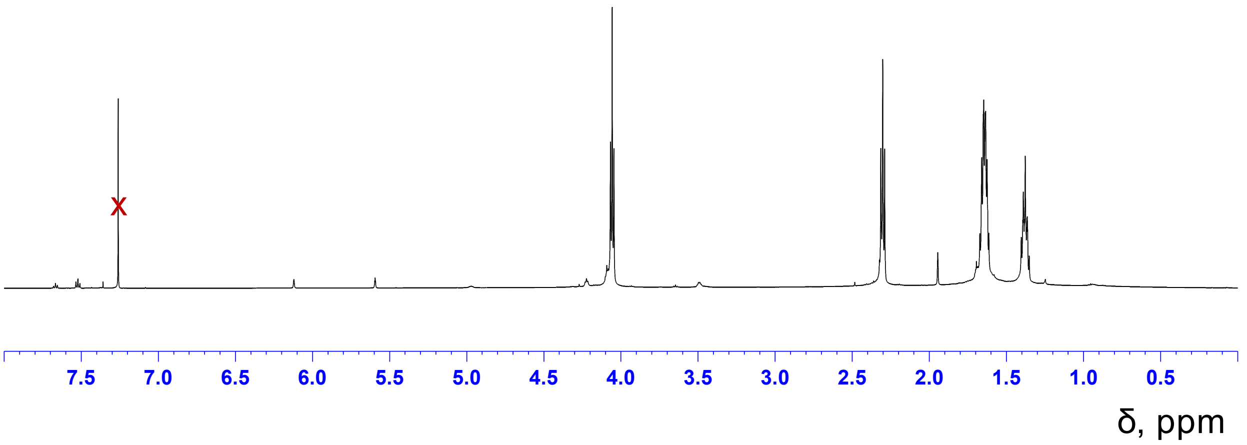

Figure S6. ^{1}H NMR spectrum rSMP recorded at 600 MHz in $CDCl_3$ at room temperature. X – solvent signal.

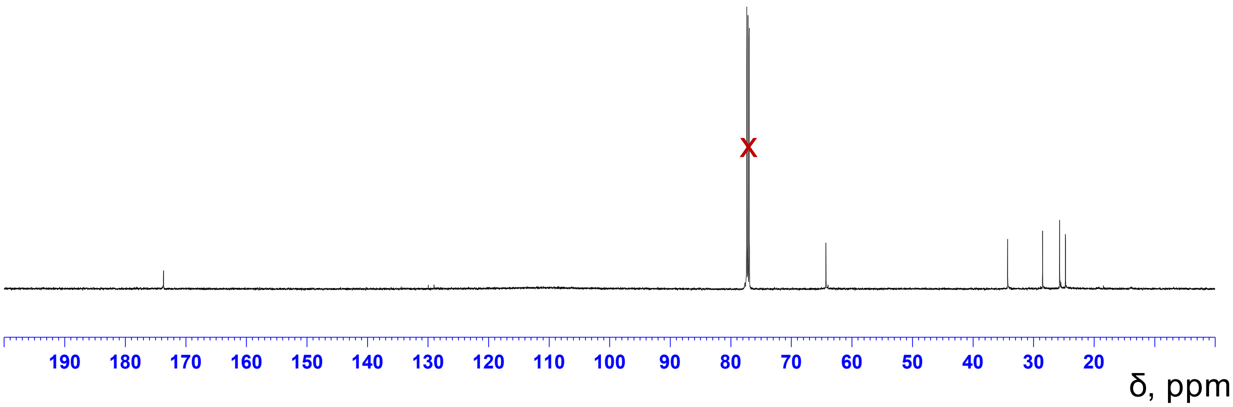

Figure S7. ^{13}C NMR spectrum of rSMP recorded at 152 MHz in $CDCl_3$ at room temperature. X – solvent signal.

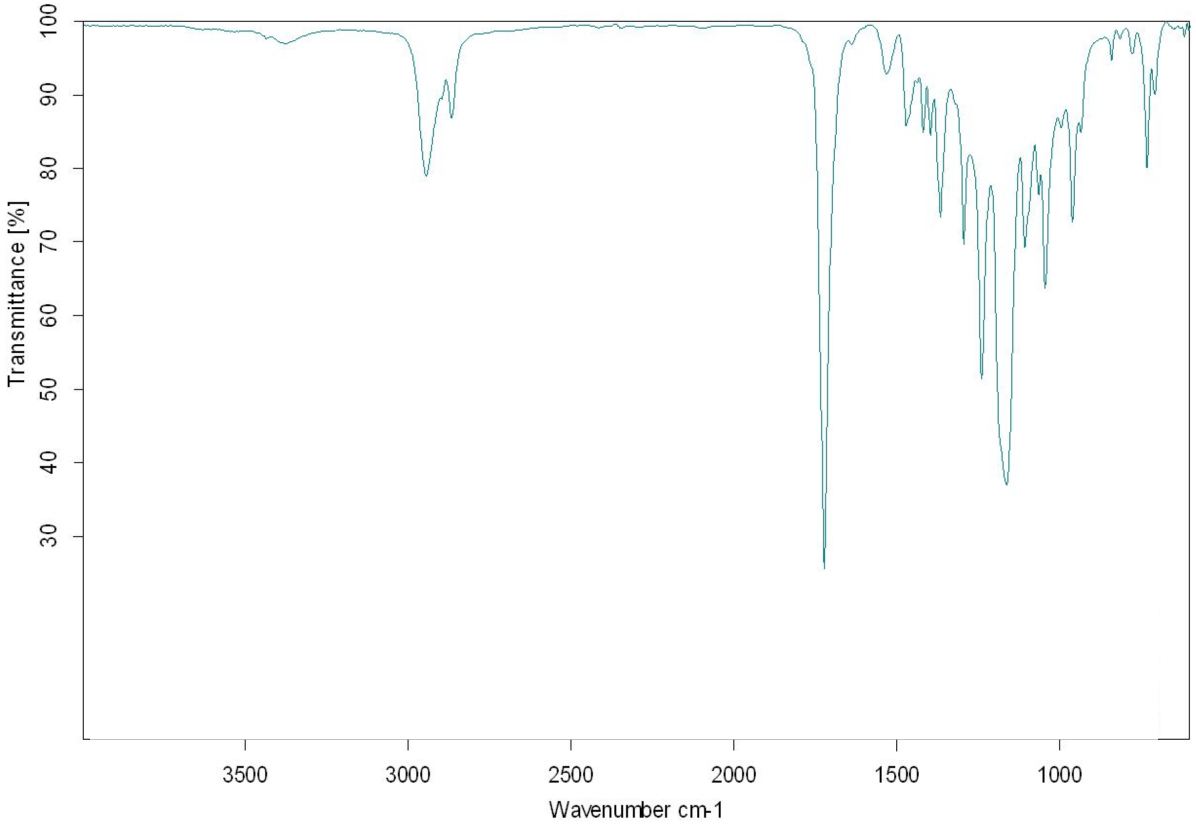

Figure S8. ATR FT-IR spectrum of rSMP.

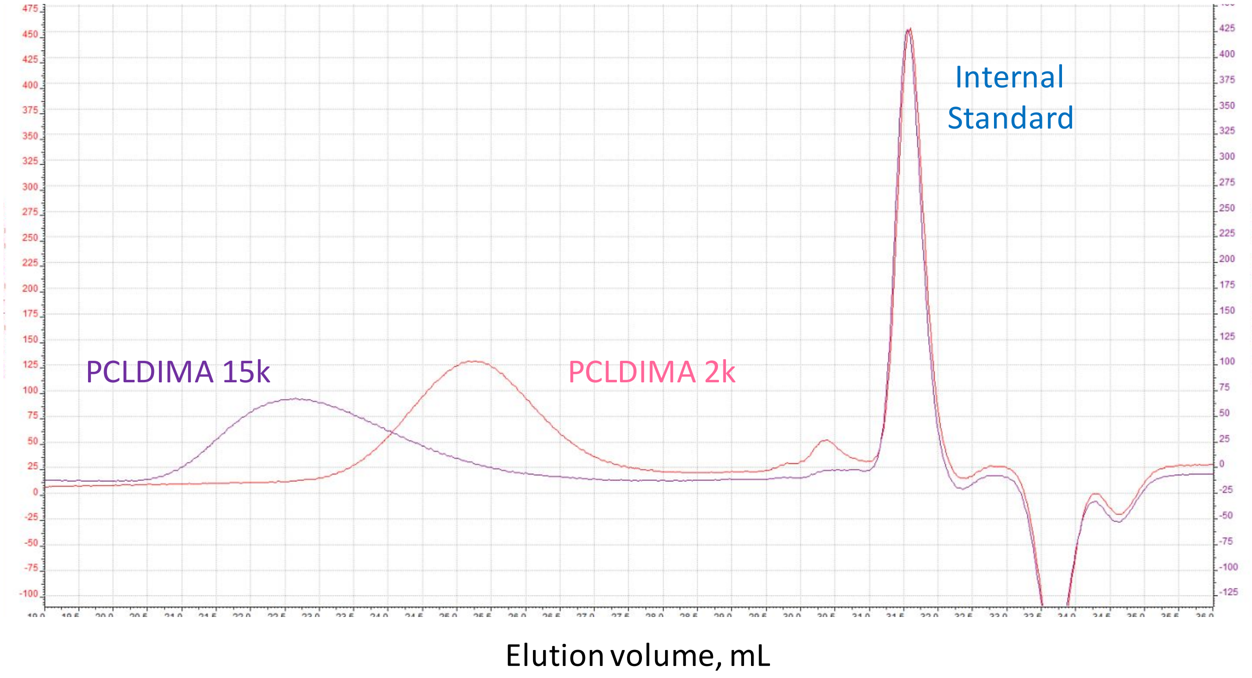

Figure S9. SEC eluograms of PCLDIMA 15k (purple trace) and PCLDIMA 2k (pink trace). THF, 40°C, 0.8 mL/min.

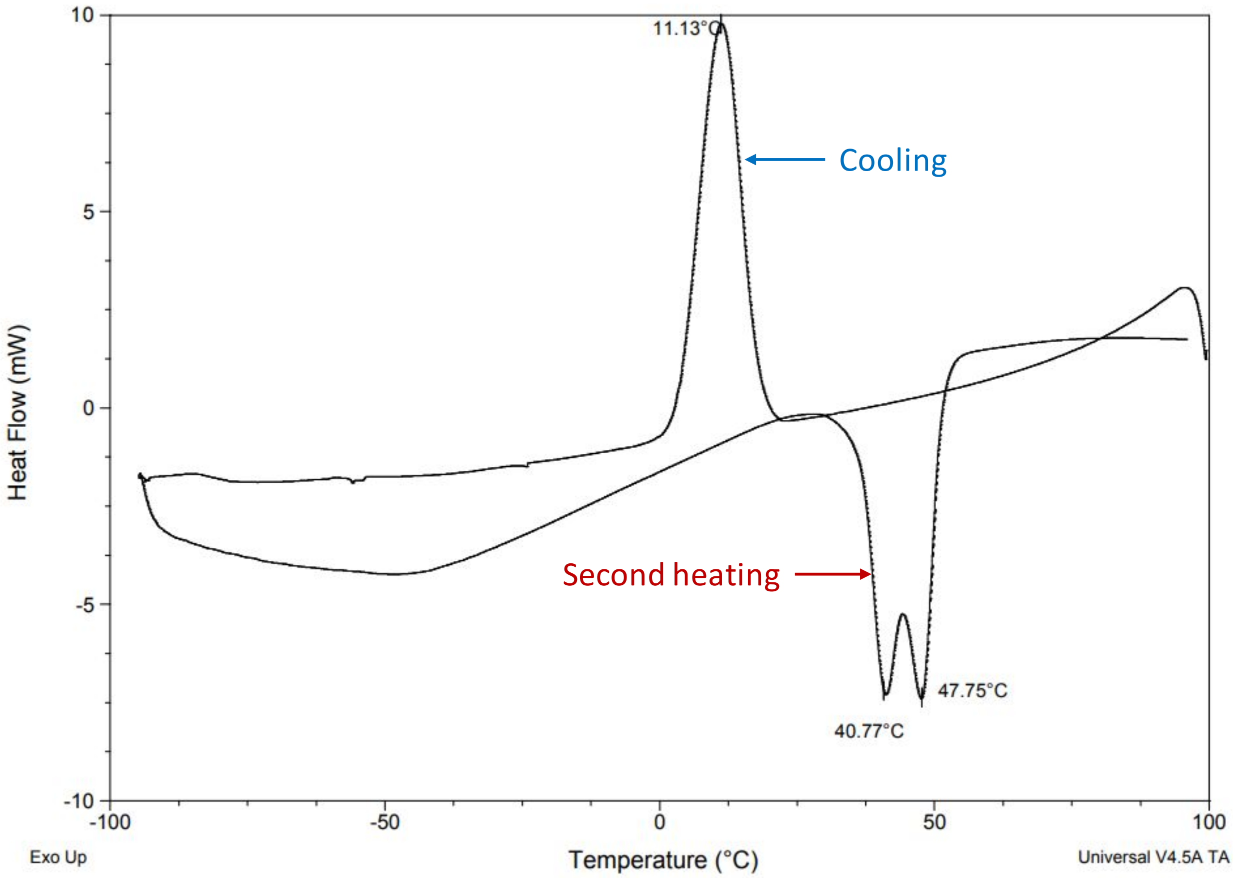

Figure S10. Thermal DSC analysis of PCLDIMA 2k. Heating/cooling rate: 10°C/min.

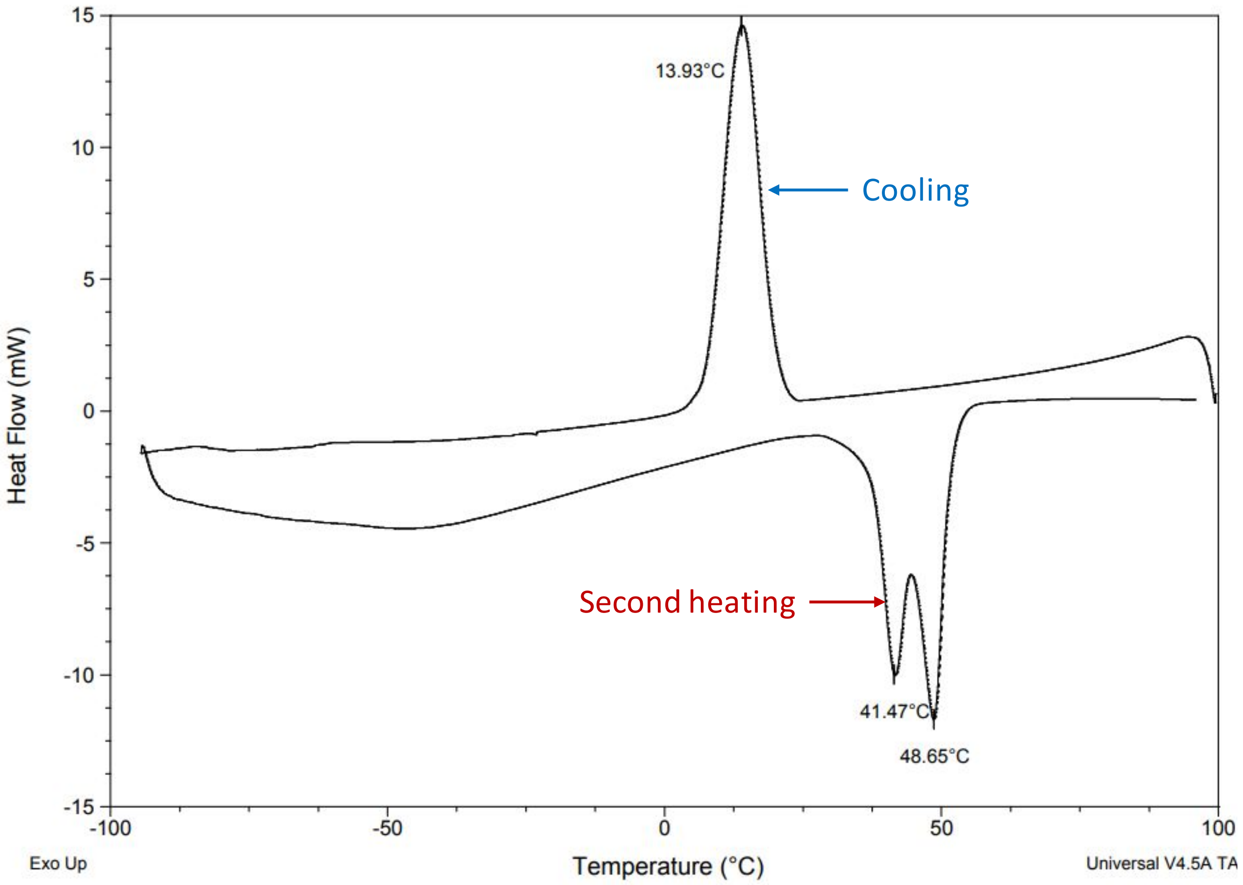

Figure S11. Thermal DSC analysis of PCLDIMA 15k. Heating/cooling rate: 10°C/min.

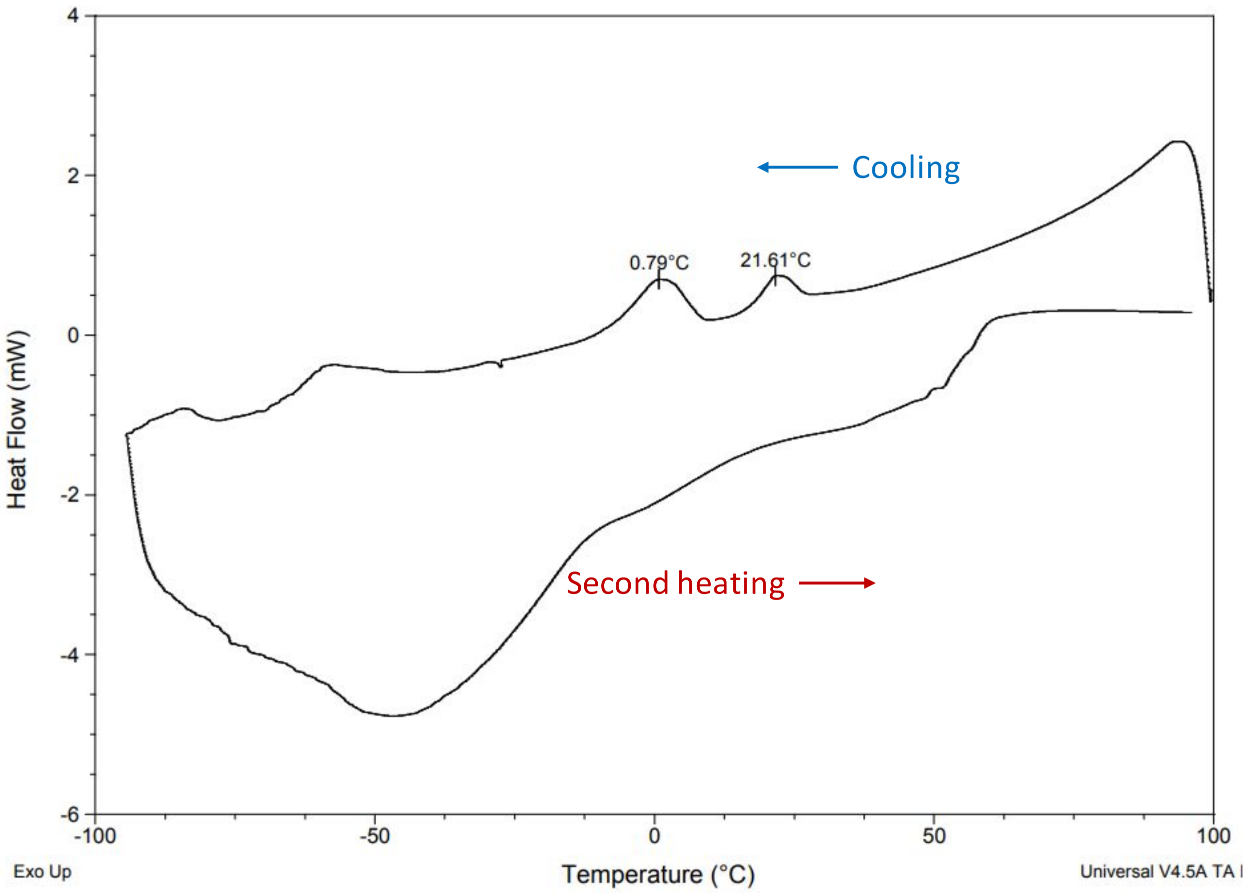

Figure S12. Thermal DSC analysis of rSMP. Heating/cooling rate: 10°C/min.
